# Outcomes, complications, and management in flow diverter treatment of cerebral aneurysms: a 15-year single-center experience

**DOI:** 10.1007/s00234-026-03979-w

**Published:** 2026-03-30

**Authors:** Emil Hikmat, Ahmet Kursat Karaman, Bora Korkmazer, Serdar Arslan, Osman Kızılkılıç, Naci Kocer, Civan Islak

**Affiliations:** 1 Department of Radiology, Medipol Bahcelievler University Hospital, Istanbul Medipol University, Istanbul, Turkey; 2 Department of Radiology, Sureyyapasa Chest Diseases and Thoracic Surgery Training Hospital, Istanbul, Turkey; 3 Division of Neuroradiology, Department of Radiology, Istanbul University Cerrahpaşa, Istanbul, Turkey

**Keywords:** Intracranial aneurysms, Flow diverters, Stents, Endovascular treatment, Complications, Secondary treatments

## Abstract

**Purpose:**

This study aimed to evaluate the complications encountered during and following the treatment of patients with flow-diverter stents, as well as the secondary interventions employed to manage these complications.

**Methods:**

We retrospectively analyzed the preprocedural, periprocedural, and postprocedural data of 660 patients with intracranial aneurysms who were treated via flow diversion between March 2008 and October 2023. Complications related to flow diversion were systematically assessed.

**Results:**

The median follow-up period was 81 months (range: 12–180 months). A total of 64 patients (9.70%) experienced at least one complication, corresponding to 65 complication events. Early complications occurred in 33 patients (5.0%), whereas late complications were observed in 31 patients (4.70%). The overall technical complication rate was 3.03%, and the clinical complication rate was 6.82%. Secondary interventions were required in 39 patients (5.91%). Procedure-related mortality and morbidity rates were 0.76% and 4.55%, respectively. Technical complications include stent shortening/migration, stent deformation, stent fracture, stent layer separation, and distal wire dissection. Clinical complications include intimal hyperplasia causing significant stenosis, stent occlusion, carotid-cavernous fistula, thromboembolic events, hemorrhagic events, and rare events such as non-ischemic cerebral enhancing (NICE) lesions and diffuse alveolar hemorrhage.

**Conclusion:**

Flow diversion represents an effective reconstructive strategy for intracranial aneurysms, demonstrating durable long-term occlusion in a large single-center cohort. Although complication patterns evolved over time, the retrospective design and concurrent changes in devices and treatment protocols preclude definitive causal inferences. This experience may serve as a practical reference for complication recognition and bailout management.

## Introduction

Since their first use in 2007, flow-diverter stents (FDS) have ushered a new era in endovascular treatment. Currently, they are the preferred method to provide parent artery reconstruction in wide-necked and complex aneurysms. The use of FDS has reduced the reliance on coiling in the treatment of wide-necked intracranial aneurysms [1]. FDS has reduced porosity compared with stents used during coil embolization, resulting in superior aneurysm coverage and therapeutic efficacy [2].

As highlighted in previous studies, branches originating from the area covered by FDSs are generally expected to remain patent [3, 4]. The reduction of blood flow into the aneurysm triggers an inflammatory reaction within the aneurysm sac, followed by thrombosis. FDSs simultaneously promote neointimal proliferation and contribute to the remodeling of the parent artery, playing a pivotal role in the healing process. This fascinating interplay of biological and mechanical responses emphasizes the elegance of endovascular treatments in addressing complex vascular conditions [3]. Unlike coil embolization, FDSs ensure gradual rather than immediate aneurysm occlusion, offering superior long-term outcomes, particularly in wide-necked aneurysms [5]. The use of flow diversion in the treatment of intracranial aneurysms is associated with a high technical success rate and a significant likelihood of complete aneurysm occlusion [6]. However, it is important to emphasize that the complication rates associated with flow diversion cannot be underestimated.

Technical challenges, such as stent shortening/migration, braid deformation, stent fracture, stent layer separation, distal wire dissection/perforation and deployment difficulties may occur. Additionally, a range of postprocedural clinical complications may occur, including stent occlusion, carotid cavernous fistula, thromboembolic complications, and hemorrhagic events. Less frequent complications have also been reported, such as non-ischemic cerebral enhancing (NICE) lesions, myocardial infarction, and diffuse alveolar hemorrhage.

Furthermore, due to the high metallic coverage of the FDS on the vessel wall, occlusion of small branches from the anterior and posterior circulation arteries, such as the lenticulostriate arteries, may occur, leading to an increased risk of ischemic stroke. There is a limited number of studies in the literature addressing complications during and after flow-diverter stent treatment, as well as the management of these complications [2, 3]. Procedure-related complications can generally be attributed to technical factors and, secondarily, to hemodynamic influences. Delayed complications; however, require consideration of more complex and less understood biological factors [7].

In the present study, we provide a comprehensive descriptive analysis of technical and clinical complications observed over a 15-year period in a large single-center cohort treated with flow diversion. Particular emphasis is placed on bailout strategies and secondary interventions. Given the temporal evolution of devices, imaging techniques, antiplatelet protocols, and operator experience, the findings should be interpreted within a descriptive and hypothesis-generating framework rather than as evidence of direct causal relationships.

## Materials and methods

### Patient selection and initial work-up

The study was approved by the institutional Ethics Committee and conducted in accordance with the principles of the Declaration of Helsinki. Written informed consent was obtained from all participants. A total of 660 patients who underwent flow diversion treatment between March 2008 and October 2023 and had complete follow-up data were included. Only patients with a minimum follow-up duration of 12 months were eligible for analysis.

Aneurysms were classified as ruptured or unruptured at presentation. Descriptive comparisons of major complications and mortality were performed according to rupture status.

## Medication and treatment

### Anticoagulation and antithrombotics

All patients were administered 75 mg/day Clopidogrel and 300 mg/day acetylsalicylic acid for at least 7 days before stent implantation. After 2010, Clopidogrel resistance test (CRT) was performed on each patient at the end of first week with dual antiplatelet medication. The patients who reached the effective platelet inhibition rate were accepted for the treatment. Clopidogrel and acetylsalicylic acid (ASA) loading was continued at the same doses for seven more days in the patients who could not reach adequate platelet inhibition. CRT was repeated at the end of seven-day period. The patients who did not reach the effective dose were accepted as “clopidogrel resistant”. Clopidogrel and acetylsalicylic acid were discontinued in this patient group and Prasugrel (10 mg/day) or Ticagrelor (90 mg 2 × 1/day) ± 100 mg/day ASA was administered for at least seven days. At the end of seven days, CRT was repeated and clopidogrel resistant patients were considered eligible for treatment at this stage.

In cases of subarachnoid hemorrhage, prior to the stent placement in the neuroangiography unit, the emergency drug loading regimen via nasogastric or orogastric tube was applied with 300–600 mg Clopidogrel + 300 mg ASA before 2014, since then following the access to the novel thienopyridines in the market, Prasugrel 40 mg or Ticagrelor 180 mg ± 100 mg ASA was administered.

During the procedure, systemic heparinization was initiated with 5,000 IU of heparin administered intravenously and continued with hourly injection of 1,000 IU of heparin to maintain an activated clotting time of 250–300 s.

Post-procedurally, heparinization continued with hourly injections of 1,000 IU for 8 h, followed by low-molecular-weight heparin (LMWH). Upon discharge, CRT proven antithrombotic treatment was maintained for at least 6 months. Following the sixth month follow-up imaging, in cases where no stent deformation or in-stent stenosis accompanying the patent stent was detected, the antithrombotic regimen was maintained permanently with ASA monotherapy 100–300 mg/day. In cases with stent deformation or in-stent stenosis, the CRT proven effective antithrombotic regimen was continued for an additional 6 months.

## Endovascular procedure: flow-diverter stent technique

All flow-diverter stent implantations were performed using Philips Integris Allura and Allura Xper FD 20/20 Biplane Angiography systems under general anesthesia. Procedures were conducted via unilateral femoral access, with a 6 F long introducer sheath and intracranial distal access catheter. The microcatheter-microguidewire system was advanced across the aneurysm segment to ensure full neck coverage before stent deployment.

Since 2010, flat-panel detector C-arm CT angiography has been used alongside standard angiography to assess stent apposition. When necessary, a second stent or balloon angioplasty was applied for optimization of stent opening and wall apposition. Control angiography was performed before concluding the procedure.

## Post-treatment follow-up

Post-procedural angiograms were obtained in anteroposterior, lateral, and working projections, followed by neurological monitoring in the intensive care unit. The standard follow-up protocol included:

- 1st Month: Neurological examination and MRI.
- 3rd and 6th Months: Flat-panel detector C-arm CT angiography.
- 12th Month: DSA and flat-panel detector C-arm CT angiography, reviewed by senior neuroradiologists.

If complete aneurysm occlusion was confirmed at 1 year, patients underwent annual clinical evaluations with further imaging as needed. For persistent aneurysm filling, annual MR angiography was performed.

A final DSA or flat-panel detector C-arm CT angiography was scheduled at year 5. Follow-up focused on stent patency, aneurysm occlusion, residual filling, and complications, with morbidity and mortality assessments.

### Definitions and event attribution

All complication rates were calculated on a per-patient basis, considering the first flow-diverter procedure performed during the study period as the index treatment. In patients undergoing retreatment or additional procedures, subsequent interventions were analyzed descriptively but were not considered independent index cases. A single patient could experience more than one complication, and a single complication could result in more than one secondary intervention.

Technical complications were defined as device- or deployment-related mechanical events occurring intra-procedurally or detected during follow-up imaging. Clinical complications were defined as neurological or systemic adverse events temporally associated with the treatment. Secondary interventions were defined as any additional endovascular or medical treatment performed specifically to manage a complication.

Non-hemodynamically significant stent deformation patterns and low-grade in-stent stenosis were not prospectively graded using standardized quantitative criteria. These findings were documented only when considered hemodynamically significant, progressive, symptomatic, or requiring secondary intervention. Therefore, the reported rates for stent deformation and in-stent stenosis reflect clinically relevant cases rather than the full spectrum of imaging-detected changes.

Complication events were recorded individually; therefore, a single patient could contribute more than one event to the analysis. When event counts are presented, totals may exceed the number of affected patients. Secondary intervention rates were calculated per patient; therefore, if multiple complication events occurred in a single patient, this individual was counted once in the secondary treatment analysis.

## Operational definitions and imaging criteria

Migration or shortening was diagnosed on digital subtraction angiography (DSA) by comparison with the immediate post-deployment angiographic images, defined as measurable displacement from the intended landing zone or inadequate aneurysm neck coverage.

Fish-mouth deformation was identified on DSA and/or C-arm CT as incomplete expansion at the proximal or distal stent edge resulting in a funnel-shaped configuration with incomplete wall apposition.

In-stent stenosis (ISS) or contrast gap was diagnosed on follow-up DSA, CTA, or MRA as luminal narrowing within the stent construct compared with the reference vessel diameter. Only hemodynamically significant stenosis or cases requiring secondary intervention were included in the complication analysis.

Stent occlusion was defined as complete absence of antegrade flow within the stented segment on DSA or CTA.

Thromboembolic events were diagnosed either angiographically (intraluminal thrombus formation during the procedure) or clinically, confirmed by diffusion-weighted MRI demonstrating acute ischemic lesions in a vascular territory consistent with the treated vessel.

Delayed aneurysmal rupture was diagnosed by non-contrast CT demonstrating subarachnoid or intracerebral hemorrhage after the index procedure, with confirmation by DSA when indicated.

Carotid-cavernous fistula was diagnosed on DSA by visualization of abnormal arteriovenous shunting between the internal carotid artery and the cavernous sinus.

Non-ischemic cerebral enhancing (NICE) lesions were diagnosed on contrast-enhanced MRI as delayed parenchymal enhancing lesions without diffusion restriction and without imaging evidence of acute infarction.

### Statistical analysis

All statistics were computed with the SPSS statistical package version 23.0. After the research data were digitized, frequency and percentage values were calculated for categorical variables, and mean, median, and standard deviation values were calculated for continuous variables.

### Era stratification

For temporal descriptive analysis, the 15-year study period (2008–2023) was stratified into three predefined treatment eras based on major institutional and technological milestones: Early Era (2008–2011), Intermediate Era (2012–2015), and Late Era (2016–2023). Era definitions reflected cumulative changes in flow diverter device technology, delivery platform miniaturization (0.027” to 0.021” to 0.017” microcatheter compatibility), integration of flat-panel C-arm CT (2010), implementation of routine platelet-function testing (2014), and progressive standardization of antiplatelet management. This stratification was performed for descriptive purposes and not for causal inference.

## Results

Among the 660 patients included in the study, 64 patients (9.70%) experienced at least one complication. A total of 65 complication events were recorded, as one patient developed two distinct late adverse events. Early complications occurred in 33 patients (5.0%), whereas late complications occurred in 31 patients (4.70%), with one patient contributing two late events. Secondary treatments were required in 39 patients (5.91%).

Of the 660 patients included in the study, 32 (4.85%) presented with subarachnoid hemorrhage at admission and underwent flow-diverter treatment. No procedure-related mortality occurred in the ruptured subgroup. Descriptively, the distribution of major complications did not demonstrate a clear increase in patients treated for ruptured aneurysms compared with unruptured cases.

Demographic and aneurysm characteristics of the patient population are summarized in Table 1. The majority of patients were female (73.33%), and the mean age was 50.9 years (range: 12–78). Aneurysms were predominantly saccular in morphology (90.9%) and located in the anterior circulation (87.9%). Most patients (88.79%) presented with a single aneurysm, and 82.7% of aneurysms measured less than 10 mm in diameter. The average aneurysm diameter was 8.73 mm (range: 1.5–57 mm).

**Table 1:** Demographic and Clinical Characteristics of Patients with Cerebral Aneurysms

Characteristic	Value	Characteristic	Value
Patients (*n* = 660)		Aneurysm Characteristics (*n* = 695)	
Gender		Morphology	
– Female	484 (73.3%)	– Saccular	632 (90.9%)
– Male	176 (26.7%)	– Dissecting	38 (5.5%)
**Age (years)**		– Fusiform	19 (2.7%)
– Mean ± Range	50.9 (12–78)	– Blister-like	6 (0.9%)
**Number per patient**		**Size**	
- Single	586 (88.79%)	– Small (< 10 mm)	575 (82.7%)
- Two	52 (7.88%)	– Large (10–25 mm)	98 (14.1%)
- Multiple	22 (3.33%)	– Giant (> 25 mm)	22 (3.2%)
**Location (aneurysm-level)**		**Diameter (mm)**	
– Proximal anterior circulation	415 (59.7%)	– Mean	8.73
– Distal anterior circulation	196 (28.2%)	– Range	1.5–57
– Posterior circulation	84 (12.1%)		

At the 6-month follow-up, the complete aneurysm occlusion rate was 79.6% among patients with available imaging. This rate increased to 85.9% at 12 months. Over the entire follow-up period, with a median duration of 81 months, the overall complete occlusion rate reached 91.8% based on the last available imaging assessment (Table 2).

**Table 2:** Complete Aneurysm Occlusion Rates According to Follow-Up Timepoint and Imaging Modality

Follow-up timepoint	Patients with available imaging (*n*)	Complete occlusion	Imaging modality
6 months	642	511 (79.6%)	DSA or flat-panel C-arm CT angiography
12 months	618	531 (85.9%)	DSA or flat-panel C-arm CT angiography
Overall follow-up (median 81 months)	564	518 (91.8%)	DSA or flat-panel C-arm CT angiography

Location-stratified analyses were additionally performed on a per-aneurysm basis to account for patients harboring multiple aneurysms in different anatomical segments. Posterior circulation aneurysms demonstrated numerically higher rates of major complications (11.9%) and secondary interventions (8.3%) compared with proximal (8.9% and 5.5%) and distal anterior circulation aneurysms (8.7% and 4.6%). Complete occlusion rates were lower in posterior circulation aneurysms (80.4%) compared with proximal anterior circulation aneurysms (95.3%). (Table 3).

**Table 3:** Complications and Secondary Interventions Stratified by Aneurysm Location (Per-Aneurysm Analysis)

Location	*N* (aneurysms)	Complications *n* (%)	Secondary Interventions *n* (%)	Overall complete Occlusion *n* (%)
Proximal anterior circulation	415	37 (8.9%)	23 (5.5%)	95.3%
Distal anterior circulation	196	17 (8.7%)	9 (4.6%)	85.5%
Posterior circulation	84	10 (11.9%)	7 (8.3%)	80.4%
Total	**695**	**64**	**39**	91.8%

Complications are categorized based on timing as peri-procedural (early) and post procedural (late) and further classified as technical or clinical (Table 4). A retrospective evaluation of the stents used in these patients’ initial treatments is presented in Table 5.

**Table 4:** Technical and clinical complication events observed in early and late periods (event-based analysis)

Technical Complications	*N*	%	Early	Late
Shortening and proximal migration of FDS	11	1.67%	5	6
Fracture of FDS	1	0.15%	1	0
Severe braid deformation (hemodynamically significant)	4	0.61%	0	4
Separation between stent layers and contrast gap	3	0.45%	0	3
Distal wire dissection	1	0.15%	1	0
Clinical Complications	*N*	%	Early	Late
Severe intimal hyperplasia causing hemodynamically significant stenosis (> 50%)	5	0.76%	0	5
Stent occlusion	10	1.52%	5	5
Carotid-cavernous fistula	3	0.45%	2	1
Non-ischemic cerebral enhancing (NICE) lesions	1	0.15%	1	0
Thromboembolic complications	16	2.42%	14	2
Hemorrhagic complications	10	1.52%	4	6
└── Distal wire perforation	2	0.30%	2	0
└── Early drug-related hemorrhage	2	0.30%	2	0
└── Delayed aneurysmal rupture	6	0.91%	0	6

*Non-hemodynamically significant stent deformation patterns and low-grade in-stent stenosis were not systematically quantified and are not included in incidence calculations.

**Table 5:** Stent Usage and Associated Complications

Stent Type	Number of implanted FDs	Percentage (%)	Migration	Deformation	Stent Occlusion	Stenosis
Silk	127	17.64%	3	1	3	1
Silk Vista Baby	20	2.78%	0	0	0	1
Fred	199	27.64%	1	0	4	2
Fred Jr.	80	11.11%	3	0	1	0
Pipeline	78	10.83%	2	1	0	0
Pipeline Flex	141	19.58%	2	1	2	0
Pipeline Vantage	26	3.61%	0	0	0	0
Pipeline Shield	4	0.56%	0	0	0	0
Surpass	15	2.08%	0	0	0	1
Derivo	15	2.08%	0	0	0	0
Derivo 2	15	2.08%	0	1	0	0

### Technical complications and stent-related issues

#### Stent migration

Secondary treatment was administered to 10 of the 11 patients (1.52%) with stent migration. In 7 patients (1.06%), a telescopic placement of an additional flow-diverter stent was used as the secondary treatment. In 3 patients (0.45%), parent artery occlusion was performed. Retrospective analysis revealed that stent migration most frequently occurred in cases where Silk and Fred Jr. stents were used.

**Fig. 1:**
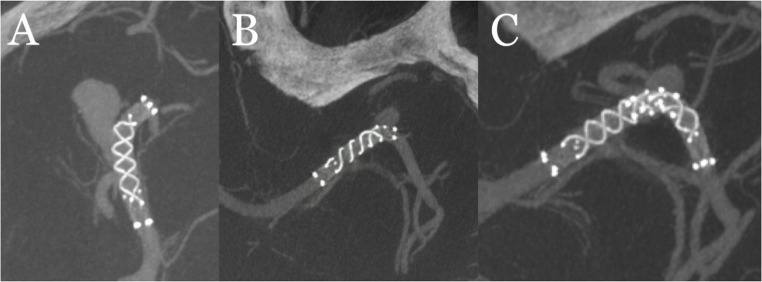
Following the FDS (FRED JR. 3×19–14 mm) treatment of an aneurysm at the left MCA bifurcation (**A**), follow-up imaging revealed distal stent shortening (**B**). As a secondary intervention, the stent was telescopically extended distally using an additional stent (**C**)

In one patient, a 9 × 6 mm laterally oriented aneurysm located at the left MCA bifurcation was treated with an FDS (FRED JR. 3 × 19–14 mm). During the 1-year follow-up flat-panel CT angiography, shortening at the distal segment of the stent was observed. As a secondary treatment, a secondary stent (FRED JR. 3 × 13–9 mm) was deployed telescopically through the initial stent to extend it distally (Fig. 1).

### Stent deformation

Stent deformation was observed in the late postoperative period, typically presenting as mild distal narrowing with a “fish-mouth” configuration. The initial flow-diverter stent treatments in these cases are detailed in Table 5. Four patients (0.61%) with stent deformation required secondary treatment; two underwent balloon angioplasty, and two received both balloon angioplasty and a secondary telescopic stent implantation.

**Fig. 2:**
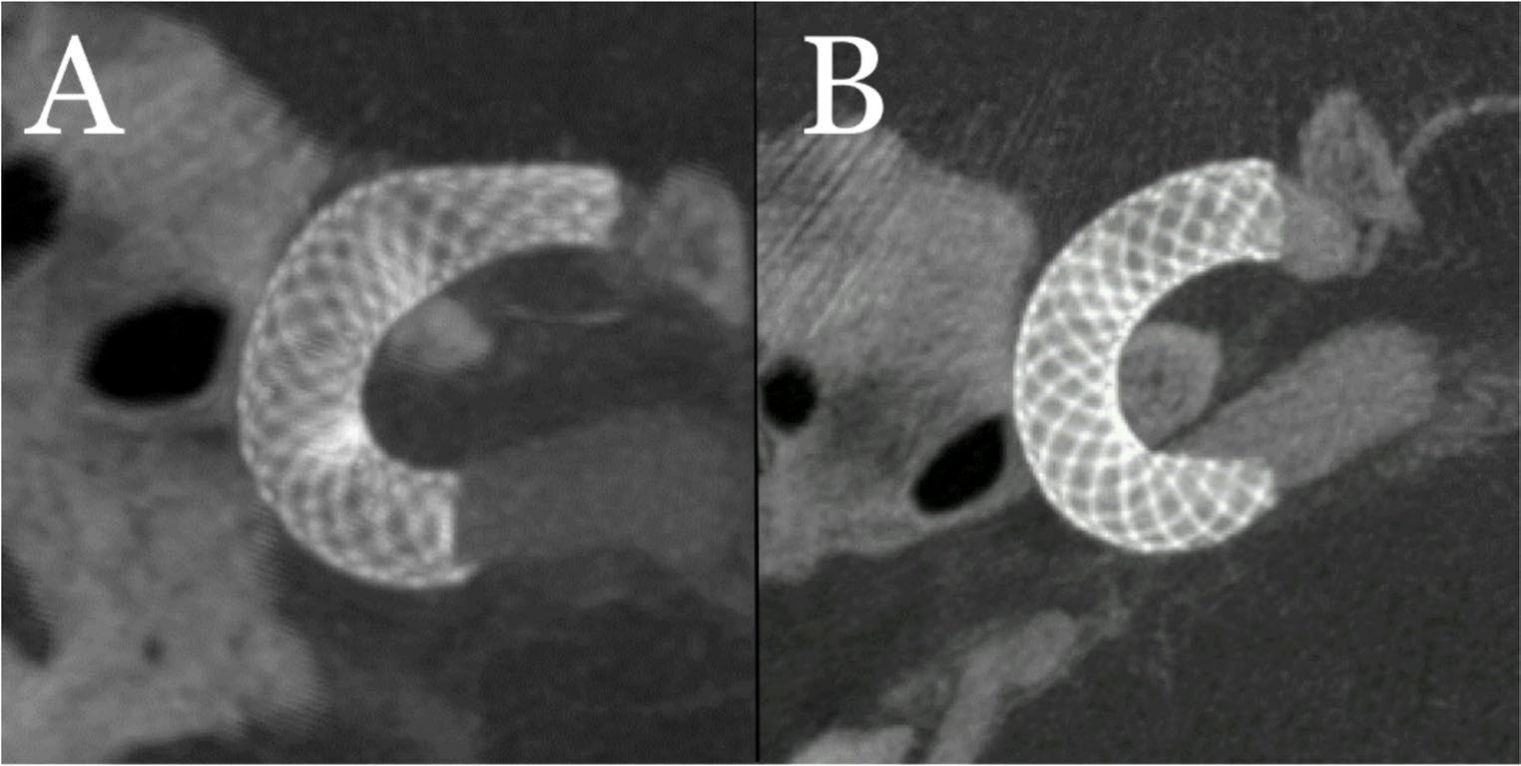
In flat-panel CT angiography, significant expansion of the FDS (Pipeline Flex 4x14mm) is observed (**B**) in a patient who underwent balloon angioplasty due to the development of fish-mouth deformity (**A**)

In one patient, a 4 × 3 mm aneurysm located in the parophthalmic segment of the left ICA was treated with an FDS. During the 1-month follow-up, a distal stent narrowing resembling a constriction (fish-mouth deformity) was detected, and balloon angioplasty was performed as a secondary treatment (Fig. 2).

### Stent fracture and dual-layer separation

Stent fracture occurred in one patient (0.15%) involving a Silk 4.5 × 25 stent in the parophthalmic ICA. Despite unsuccessful attempts at stent retrieval or apposition, intra-procedural flow remained preserved. Follow-up imaging demonstrated delayed ICA occlusion at 9 months, with partial recanalization confirmed on DSA at 5 years.

The FRED stent has a unique dual-layer mesh design, consisting of a low-porosity inner layer and a high-porosity outer layer. This dual-layer system enhances scaffolding effects and facilitates full fluoroscopic visualization of the stent [8]. In three patients (0.45%) treated with FRED stents, a contrast gap along the stent wall was observed post-procedurally. In two cases, this was attributed to separation between the inner and outer layers of the dual-layer FRED stent, while in one case the gap occurred between telescopically placed stents (Silk and Leo). In a case with FRED stent inner layer separation, a telescopically deployment of another FDS was performed as a secondary intervention.

### Iatrogenic dissection

In one patient (0.15%), iatrogenic dissection caused due to the distal wire occurred during the procedure, and the stent was successfully placed using a different microcatheter-microguidewire combination.

### Clinical complications

Clinical complications were observed in 45 of the 660 patients included in our study (6.82%) during early and late periods. The list of technical and clinical complications is presented in Table 4.

### Thromboembolic events and ischemic complications

Thromboembolic events were the most common clinical complications observed during both early and late postoperative periods. A total of 16 patients (2.42%) experienced thromboembolic complications, with 14 cases (2.12%) occurring in the early period and 2 cases (0.30%) in the late period. Stent occlusion was observed in 10 patients (1.52%), evenly split between the early and late periods (5 cases each).

Among 14 patients (2.12%) with peri-procedural thromboembolic events, IV tirofiban was administered, resolving thrombus in 7 cases. The remaining 7 patients required secondary treatment with aspiration thrombectomy, stent-retriever thrombectomy, balloon angioplasty, and telescopic placement of an additional stent. Balloon angioplasty was applied to 3 patients, and mechanical thrombectomy was performed in another 3, achieving full recanalization in all cases. In 1 patient where thrombus resolution could not be achieved, an additional stent was placed.

Acute ischemic infarction occurred in two patients during the peri-procedural period. Both patients demonstrated clinical improvement during follow-up and had a long-term modified Rankin Scale (mRS) score of 1.

Secondary treatment was performed in five patients (0.76%) with stent occlusion. One underwent balloon angioplasty, another received mechanical thrombectomy, and a third required an additional FDS after failed angioplasty. In a case of stent migration and occlusion, IV tirofiban and stentectomy restored the flow. One patient with late occlusion improved with dual antithrombotic therapy, showing recanalization at one year.

### In-stent stenosis

During the late postoperative period, follow-up imaging revealed in-stent intimal hyperplasia caused by neointimal proliferation, leading to varying degrees of luminal narrowing within the flow-diverter stent and the development of in-stent stenosis.

In our cohort, secondary intervention was required in five patients with in-stent stenosis exceeding 50%. These patients were treated with balloon angioplasty, which resulted in favorable outcomes in all cases. Follow-up DSA imaging demonstrated a positive response to the treatment and regression of in-stent stenosis in the long-term.

#### Hemorrhagic complications

**Fig. 3:**
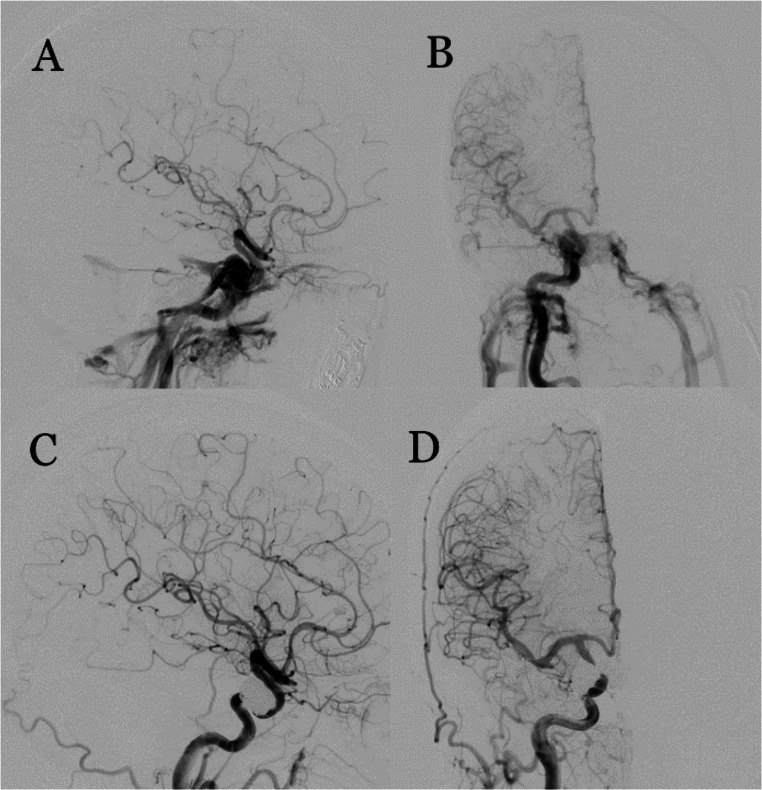
(**A**,**B**) Lateral and AP angiographic views of a patient with a right ICA cavernous segment aneurysm, showing delayed carotid-cavernous fistula (CCF) formation with contrast passage into the cavernous sinus and venous system following the flow diversion with FRED 5.5x32-26mm. (**C**,**D**) Post-transvenous coil embolization, angiographic images in lateral and AP views demonstrate complete resolution of the fistula

Hemorrhagic complications occurred in 10 patients (1.52%) and were classified as procedure-related, drug-related, or delayed aneurysmal rupture. Procedure-related hemorrhages occurred in two patients (0.30%) due to distal wire perforation, managed with heparin reversal and proximal balloon occlusion. Drug-related hemorrhages were observed in two patients (0.30%): one had an early contralateral intraparenchymal hematoma likely due to antithrombotic therapy; the other developed diffuse alveolar hemorrhage, temporally associated with combined anticoagulation and dual antiplatelet therapy, which resolved after cessation of heparin. Delayed aneurysmal rupture occurred in six patients (0.91%). In one patient, rupture of a large cavernous aneurysm at two months resulted in carotid-cavernous fistula formation, which was successfully treated with transvenous coil embolization (Fig. 3).

### Carotid-cavernous fistula

Carotid-cavernous fistula developed in three patients (0.45%). Two cases occurred intraoperatively due to iatrogenic laceration of the cavernous ICA during stent deployment. One patient was treated via transvenous coil embolization, while the other underwent transarterial coil embolization via the lacerated ICA segment as secondary intervention. The third case developed in the late postoperative period, approximately two months after treatment, due to delayed rupture of a large cavernous segment aneurysm, likely triggered by thrombus load–induced proteolytic vessel wall degradation. This patient was also successfully treated with transvenous coil embolization. Importantly, this case corresponds to the patient described under delayed aneurysmal rupture, representing two related but distinct late complication events within the same individual.

#### Rare clinical complications

**Fig. 4:**
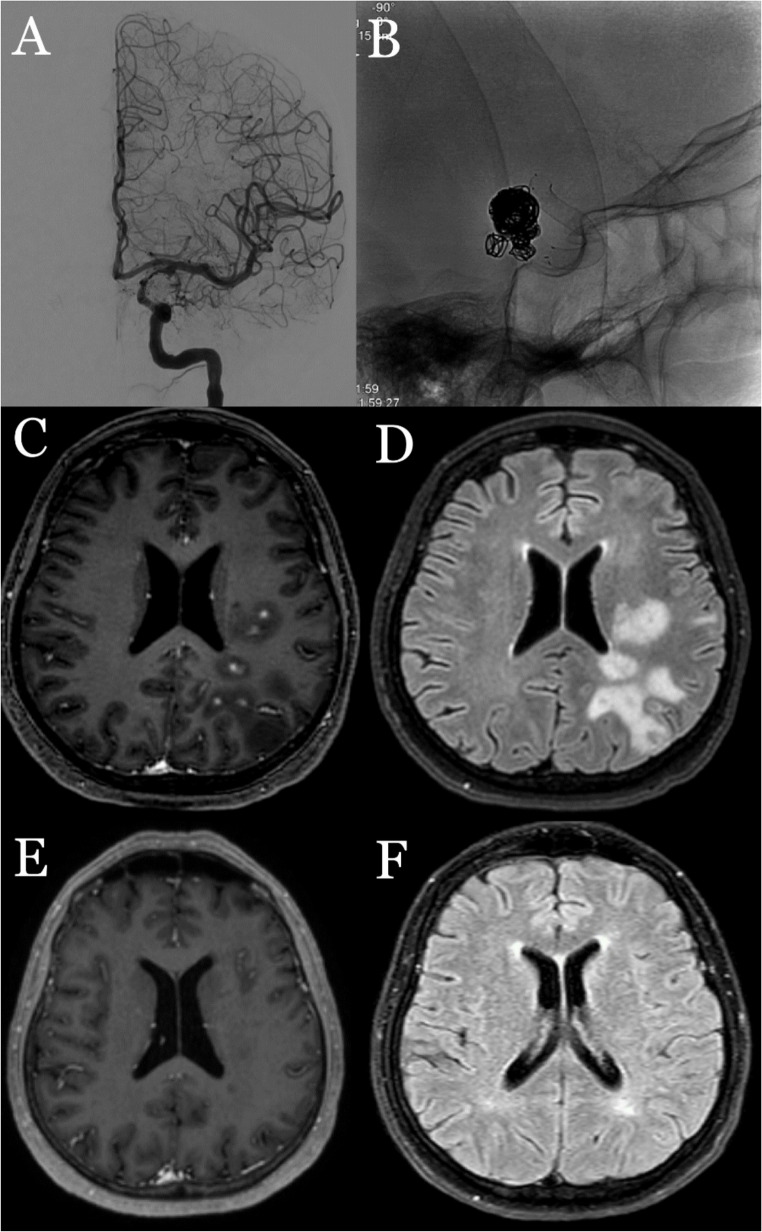
(**A**,**B**) Endovascular embolization with a flow-diverter stent (Derivo 4.5x20mm) and coil performed for two aneurysms in the left ICA. (C, D) MRI obtained 2.5 weeks post-procedure shows nodular lesions with contrast enhancement in the left hemisphere, surrounded by areas of vasogenic edema. (**E**, **F**) Follow-up MRI after treatment demonstrates regression of the NICE lesions and the surrounding vasogenic edema

A patient experienced headache, focal right arm convulsions, aggressive behavioural changes 18 days after a successful flow diversion (Derivo 4.5 × 20) for a left ICA parophthalmic aneurysm. CT revealed vasogenic edema, and MRI showed contrast-enhancing nodular lesions with surrounding edema in the treated hemisphere, suggesting non-ischemic cerebral enhancing (NICE) lesions. The patient was started on 1 g/day intravenous methylprednisolone (MPS) treatment for 7 days, then followed with maintenance oral MPS. This treatment led to near-complete symptom resolution, and the patient was discharged with total regression of neurological findings (Fig. 4).

Additionally, one patient developed an early postoperative myocardial infarction. Given the absence of clear procedural causality, this event was reported descriptively but excluded from the core neurovascular complication rate.

#### Morbidity and mortality

Among 660 patients treated with flow-diverter stents for intracranial aneurysms, a total of 5 mortalities were recorded, resulting in a procedure-related mortality rate of 0.76%. Neurological morbidity, defined as an increase in the pre-procedure mRS score or mRS ≥ 2, was identified in 30 patients during follow-up clinical examinations, corresponding to a procedure-related morbidity rate of 4.55%. The overall morbidity and mortality rate for intracranial aneurysm treatment with flow-diverter stents in our series was calculated as 5.3%.

Importantly, all mortalities were associated with hemorrhagic complications. Of the 10 patients (1.52%) who experienced hemorrhagic events, one death occurred among four early post-procedural hemorrhages, while four deaths were recorded among six patients with delayed aneurysmal rupture.

## Discussion

In this study, technical and clinical complications, along with secondary treatments performed in response to these events, were systematically analyzed in 660 patients treated with FDSs at our institution. Periprocedural and postoperative complications were evaluated to identify factors associated with treatment success and to inform strategies aimed at improving patient outcomes.

In our study, 64 of the 660 patients (9.70%) experienced at least one complication, accounting for a total of 65 complication events. Early complications occurred in 33 patients (5.0%), while late complications were observed in 31 patients (4.70%), with one patient experiencing two late events. Secondary treatment was required in 39 patients (5.91%) (Table 6). By comparison, a large meta-analysis reported an overall complication rate of 17.0% in the literature [4]. Complications related to flow diversion were classified as peri-procedural (early) or post-procedural (late) and further categorized as technical or clinical. Technical complications occurred in 20 patients (3.03%) across both early and late periods. Reported rates of technical complications in the literature vary widely, ranging from 3.1% to 33.3%, largely depending on how technical complications are defined [5]. Most of these complications do not result in clinically significant sequelae [9].

**Table 6:** Secondary interventions by complication category (event totals vs. patients treated)

Complication	Total *N*	Patients Requiring Secondary Treatment (*N*)	Details of Secondary Treatment
Shortening and proximal migration of FDS	11	10	7 patients received an additional FDS 3 underwent parent artery occlusion
Deformation of FDS	4	4	2 patients underwent balloon angioplasty 2 had balloon angioplasty plus an additional stent
Separation between stent layers and contrast gap	3	1	1 patient required a telescoped FDS
Distal wire dissection	1	1	An additional stent was deployed due to the occurrence of arterial dissection during the procedure
Intimal hyperplasia causing stenosis	5	5	All 5 patients were treated with balloon angioplasty
Stent occlusion	10	5	1 balloon angioplasty 1 mechanical thrombectomy 1 additional FDS after failed angioplasty 1 “stentectomy” + tirofiban (for migration + occlusion) 1 improved on dual antithrombotic therapy
Carotid-cavernous fistula	3	3	2 transvenous coil embolizations 1 transarterial coil embolization
Non-ischemic cerebral enhancing (NICE) lesions	1	1	Treated with steroid therapy
Thromboembolic complications	16	7	Balloon angioplasty in 3 patients (complete recanalization) Mechanical (aspiration or stent-retriever) thrombectomy in 3 patients (complete recanalization) 1 patient received an additional telescoped stent after unresolved thrombus
Hemorrhagic complications	10	3	1 patient with delayed aneurysmal rupture - transvenous coil embolization 2 patient with distal wire perforation - managed with heparin reversal and proximal balloon occlusion

Footnote: Patients may appear in more than one complication category (e.g., delayed aneurysmal rupture with secondary carotid-cavernous fistula); therefore, category-specific counts may sum to more than the total number of patients requiring secondary intervention (*n* = 39)

In terms of secondary treatments for complications after FDS placement, there are relatively few studies in the literature, making meaningful comparison challenging [10–13]. Future larger-scale, multicenter studies examining secondary treatment strategies and outcomes will be crucial in guiding clinical practice and improving overall outcomes.

Stent migration is a rare but clinically relevant complication after flow-diverter stent (FDS) implantation. Insufficient stent apposition may impair aneurysm thrombosis and endothelial healing while increasing the risk of thromboembolic events and mechanical instability, potentially resulting in migration and incomplete aneurysm neck coverage. Migration may occur due to spontaneous device shortening, parent vessel–stent diameter mismatch exceeding 1 mm, reduced wall adherence in wide-neck aneurysms, suboptimal apposition—particularly in the absence of adjunctive balloon angioplasty—and distal displacement related to in-stent microthrombus formation [6]. Importantly, migration has been reported not only as an intra-procedural event but also as a delayed phenomenon, occurring up to 14 months after treatment [6]. In the patients included in our study, late-stage stent migration was not observed. While proximal migration is more common than distal migration, it is less likely to cause neurological deficits [6].

In the literature, Zhou et al. reported a stent migration rate of 5.8% in a systematic review encompassing 60 studies, whereas Maus et al. observed intra-procedural migration in 2.2% of 46 flow-diverter cases [14]. In our series, stent migration occurred in 11 patients (1.67%), demonstrating a comparable incidence to previously reported data. Secondary endovascular treatment was required in 10 of these patients, most commonly using reconstructive strategies such as telescopic deployment of an additional flow-diverter stent, balloon-assisted repositioning, or stent-assisted coiling; parent artery occlusion was considered in selected cases with sufficient collateral circulation when reconstructive strategies were not feasible [15].

In the present study, we focused specifically on severe stent deformations that were hemodynamically relevant and required secondary endovascular treatment, rather than attempting to quantify the overall incidence of all deformation patterns. Previous work by Popica et al. reported that fish-mouthing and braid collapse were significantly associated with higher morbidity, particularly in cases requiring retreatment [16].

In our cohort, secondary endovascular treatment was required in four patients with hemodynamically significant fish-mouth deformity and braid deformation, managed with balloon angioplasty alone or balloon angioplasty combined with telescopic implantation of an additional flow-diverter stent, resulting in favourable angiographic reconstruction and no new permanent neurological deficits. However, deformations that were not classified as clinically or angiographically relevant were not systematically captured, and other deformation patterns such as braid collapse, foreshortening and braid bump were not prospectively graded according to the recently proposed “F2B2” framework [17]. This likely led to an underestimation of the overall deformation burden and represents an important limitation of our retrospective study; a more detailed, subtype-based analysis focusing the F2B2 classification is planned in subsequent work.

Beyond classification frameworks, the mechanical behavior underlying deformation patterns may also be influenced by intrinsic platform characteristics, including stent material composition and architectural design [18]. Cobalt–chromium–based constructions generally provide higher radial force and longitudinal stiffness, which may enhance wall support but can also influence foreshortening or migration dynamics in cases of diameter mismatch [19]. In contrast, more flexible nitinol-based platforms offer improved conformability in tortuous anatomy, yet may be more susceptible to deformation patterns under certain deployment conditions [20]. These considerations are offered to provide practical insight into platform behavior and should not be interpreted as evidence of device-level superiority.

During endovascular treatment, distal wire dissection or perforation can occur during microcatheter exchange over an exchange guidewire. Distal wire perforation occurred in 2 patients (0.30%) during the procedure, leading to extravasation. Temporary balloon occlusion was performed in these cases to control bleeding. Similarly, Pistocchi et al. reported an arterial perforation in the distal MCA caused by a distal wire during the placement of a Silk flow-diverter stent in a series of 30 patients. This complication was successfully managed with coil embolization as a secondary treatment, with no residual sequelae [21].

Aneurysm thrombosis after flow-diverter stent implantation occurs through two main mechanisms: conversion of intra-aneurysmal flow from turbulent to stagnant, promoting thrombosis, and progressive endothelialization across the aneurysm neck, resulting in permanent healing. Endothelialization involves mechanical injury, intimal hyperplasia, and healing. Neointimal proliferation may cause in-stent stenosis (ISS); although intimal hyperplasia is the primary mechanism, dual antithrombotic therapy may reduce early endothelial injury and platelet aggregation, thereby lowering the risk of thrombosis and stenosis. Platelet-mediated inflammatory responses further contribute to smooth muscle cell proliferation and stenosis formation.

In-stent stenosis (ISS) remains an incompletely characterized complication of FDS treatment, with widely variable reported incidence across devices. Reported ISS rates range from 6.3% to 57% for Silk stents [22, 23], 38.9% for the Pipeline Embolization Device 31% for p64, and 13.3% for Surpass stents [24, 25]. This heterogeneity is primarily attributed to differences in ISS definitions, grading criteria, and follow-up protocols [26].

In our cohort, ISS related to intimal hyperplasia was most frequently observed in segments treated with FRED stents; however, minor or borderline luminal narrowing may have been under-recognized, as such changes were not the primary focus of routine follow-up reporting. ISS management was guided by functional significance rather than angiographic appearance alone. Secondary endovascular treatment was performed in five patients with hemodynamically relevant or progressive stenosis using balloon angioplasty, with follow-up DSA demonstrating lumen widening, regression of stenosis, and no permanent neurological deficits. Because standardized ISS grading and routine quantitative lumen measurements were not applied, an overall ISS incidence was not reported, and the analysis focused on clinically significant cases requiring secondary endovascular treatment.

In our clinic, dual antithrombotic therapy is administered for six months after treatment, regardless of imaging evidence of ISS, followed by lifelong aspirin monotherapy (100–300 mg) in the absence of stenosis.

As endovascular treatment of intracranial aneurysms becomes more widespread, the risk of thromboembolic complications increases due to the thrombogenic nature of the coils and stents used in these procedures. The primary mechanism underlying thrombosis in these complications is thought to be platelet activation, as numerous studies have demonstrated that dual antithrombotic therapy significantly reduces the risk of thromboembolism. In recent years, several studies have examined whether prolonged dual antiplatelet therapy confers additional benefit after stent-assisted aneurysm treatment. Ozaki et al. reported in a randomized multicenter trial that extending DAPT beyond three months did not significantly reduce delayed ischemic stroke, with overall event rates remaining exceedingly low in both groups [27]. Likewise, Enomoto and colleagues demonstrated in a large cohort of SAC and flow-diverter cases that patients receiving short-duration DAPT (< 90 days) experienced similar rates of thromboembolic and major bleeding complications compared with those maintained on longer regimens, provided that the early periprocedural period was uneventful [28]. However, both studies were conducted primarily in East Asian populations, where pharmacogenetic factors—particularly CYP2C19-associated clopidogrel metabolism—may influence the ischemia–bleeding balance. Therefore, although current evidence suggests that shorter DAPT courses may not increase major ischemic risk, validation in broader, racially and geographically diverse multicenter cohorts remains necessary.

Among postoperative complications, the incidence of ischemic stroke and perforator infarction has been reported as approximately 6% and 3%, respectively [24]. Very late ischemic complications (≥ 4 months) are rarely encountered in the literature [29].

In our clinic, IV tirofiban (Aggrastat) is preferred for intra-procedural thrombosis treatment due to its rapid achievement of the desired effect. In our study, IV tirofiban was administered to 14 patients (2.12%) with thrombus formation during the peri-procedural early period, achieving complete intra-procedural recanalization in 7 cases.

One of the factors that increases the risk of thromboembolism in the post-treatment period is smoking. Significant scientific studies have demonstrated a strong dose-response relationship between smoking and the risk of stroke and thrombosis. Smoking increases the risk of ischemic stroke by 3 to 4 times. It is crucial to emphasize to all patients the importance of quitting or reducing smoking after treatment and to support them through a tailored smoking cessation program [30].

In our study, one patient who underwent endovascular treatment with a Fred stent (3.5 × 22–16 mm) for a right MCA aneurysm developed hemiplegia six years later. DSA imaging revealed total occlusion of the stent. Upon questioning, the patient admitted to discontinuing ASA 300 therapy and resuming smoking. Dual antithrombotic therapy was initiated, and follow-up angiographic examinations one year later showed recanalization. The patient had a mRs score of 1 at the last follow-up.

It is noteworthy that early clinical series reported a thromboembolic complication rate approaching 15%, reflecting the initial learning curve, use of multiple overlapping devices, and lack of standardized platelet reactivity testing. However, over time, complication rates declined—as evidenced by a multicenter study showing a drop from 15.8% during 2011–2013 down to 8.9% in 2018–2019—concurrent with refinements in patient selection, device deployment protocols, routine antiplatelet responsiveness assessment, and growing operator experience. These factors collectively contributed to a steep reduction in both acute and delayed thromboembolic events in contemporary practice [31].

Hemorrhagic complications following flow diverter treatment can be categorized into three main groups: procedure-related, drug-related, and delayed aneurysmal ruptures. Procedure-related hemorrhages include intraoperative events such as distal wire perforation, which may lead to subarachnoid or parenchymal bleeding. Drug-related hemorrhages typically occur in the early postoperative period and may present as intracranial hemorrhages associated with dual antiplatelet therapy, or less commonly, as diffuse alveolar hemorrhage. Delayed aneurysmal rupture, although rare, remains the most severe among the hemorrhagic complications, particularly in large or giant aneurysms, and is associated with high morbidity and mortality.

Intracerebral hematoma (ICH) is one of the most feared complications of aneurysm treatment with FDSs. While rarely reported in stent-assisted coil embolization therapies, studies have shown that this risk occurs in 2–4% of cases treated with FDSs [32].

Delayed aneurysm rupture was frequently reported during the initial years of FDS use, particularly in large or giant aneurysms treated solely with FDSs, occurring within the first 6 months and often associated with high mortality rates. Since mid-2011, in our clinic, FDSs have been used in conjunction with coil embolization for treating large/giant aneurysms or aneurysms with daughter sacs, and no cases of delayed aneurysm rupture have been observed since. Delayed aneurysm rupture is a serious complication, particularly in giant aneurysms. While its etiology remains unclear, hemodynamic changes and thrombus-associated proteolytic activity are potential contributing factors [33, 34]. Other risk factors for delayed aneurysmal hemorrhages include: a-)Large and giant aneurysms b-)Symptomatic aneurysms c-)Saccular aneurysms with an aspect ratio > 1.6 d-)Late migration of the FDS into the aneurysm sac d-)Mechanical damage during FDS implantation [35–37].

A literature review on delayed hemorrhagic complications following FDS treatment indicates that 80% of these complications have poor clinical outcomes and approximately 80% occur within the first 30 days post-treatment [38]. In our study, mortality was observed in 5 out of 10 patients who developed hemorrhagic complications. Of these, one patient experienced an early drug-related hemorrhage, while the remaining four cases were due to delayed aneurysmal rupture. If the aneurysm targeted for endovascular treatment is located in the cavernous ICA segment, vascular damage during FDS placement or delayed aneurysm rupture post-stenting may lead to iatrogenic carotid-cavernous fistula (CCF).

Direct carotid-cavernous fistula (Barrow type A) following FDS treatment is rarely reported in the literature and is most commonly associated with the treatment of cavernous ICA aneurysms [33, 39–43]. Treatment of CCFs caused by FDS placement typically involves conventional methods such as transvenous embolization, parent artery occlusion, or surgical ligation [40].

However, transarterial embolization techniques for the cavernous sinus may be limited due to the presence of an FDS in the cavernous ICA. The stent may obstruct transarterial access to the rupture site where the fistula has formed.

The use of secondary FDSs in the treatment of iatrogenic carotid-cavernous fistulas (CCFs) has been reported in a few cases [43, 44]. In most of these cases, transarterial placement of FDSs was combined with ipsilateral transvenous embolization. Prior to such complex endovascular procedures, a balloon test occlusion of the ipsilateral ICA is essential to ensure procedural safety.

In our study, secondary interventions were performed to treat CCFs in 3 cases that developed during or the following the flow diversion. Two cases were successfully treated with transvenous coil embolization, while one was treated with transarterial coil embolization through the lacerated segment, achieving successful endovascular repair.

Rare non-ischemic cerebral enhancing (NICE) lesions, associated with delayed granulomatous inflammation, have been reported among FDS complications, alongside more common thromboembolic events. These lesions appear as punctate, nodular, or ring-enhancing abnormalities in the relevant vascular territory, possibly with perilesional edema. Biopsy evidence suggests that NICE lesions stem from granulomatous foreign body reactions to microemboli caused by hydrophilic polymer coatings on endovascular devices [45, 46]. Earlier hypotheses linking NICE lesions to nickel allergy are no longer supported [47], as they occur more often with cobalt-chromium rather than nickel-titanium FDSs.

In our study of 660 patients, the incidence of foreign body reaction, specifically symptomatic NICE lesions, was determined to be 0.15%. In comparison, Nakagawa et al. reported an incidence of 2.3% in a series of 305 patients, while Ikemura et al. found a 0.9% incidence in a cohort of 1754 patients undergoing coil embolization [48, 49].

A multicenter study by Richter, Cindy et al. indicates that NICE lesions occur more frequently with FDSs than other endovascular therapies, possibly due to variations in material, wire count, and mesh angle affecting stent friction and polymer microembolization [50]. Recently, polymer-based surface coatings (e.g., phosphorylcholine, glucan-based hydrophilic, biopassive polyacrylate, and fibrin-based nanocoatings) have been developed to reduce device thrombogenicity [50].

Diffuse alveolar hemorrhage (DAH) is another rare hemorrhagic complication that may occur following FDS treatment. DAH is thought to be associated with antiplatelet therapy and has primarily been reported in patients receiving glycoprotein IIb/IIIa inhibitors in previous studies [51–53]. In a study by Ali et al., involving 1020 patients treated with glycoprotein IIb/IIIa inhibitors for cardiovascular disease, the incidence of DAH was reported as 0.68% [51]. In the neurointerventional literature, post-endovascular DAH has only been reported once, as a case report [54]. Similarly, in our study, 1 patient developed DAH following the procedure. Low molecular weight heparin therapy was discontinued, and the patient was managed solely with dual antithrombotic therapy. By the 8th day of treatment, the patient was discharged with stable oxygen saturation on room air.

Brinjikji et al., in a recent meta-analysis of 29 studies, reported a procedure-related mortality rate of 4% and a morbidity rate of 5% [55]. In our study, the overall mortality and morbidity rates were 0.76% and 4.55%, respectively, aligning with findings from the literature. The total morbidity-mortality rate in our study was calculated as 5.3%. A meta-analysis of all studies in the literature found a general morbidity rate of 6.2% (95% CI, 4.7–8.1%) and a general mortality rate of 3.4% (95% CI, 2.4–4.7%) [56]. No ischemia-related mortality or permanent neurological deficit was observed, distinguishing our series from prior reports in which ischemic events contributed more prominently to morbidity. These findings align with literature indicating that delayed hemorrhagic events, although rare, are the leading cause of death in patients undergoing flow-diverter treatment.

Although patients presenting with subarachnoid hemorrhage are generally considered to carry higher baseline procedural risk, our cohort did not show an evident increase in flow-diverter–related mortality or major complication burden in this subgroup. However, the relatively small number of ruptured cases limits the strength of this observation.

**Fig. 5:**
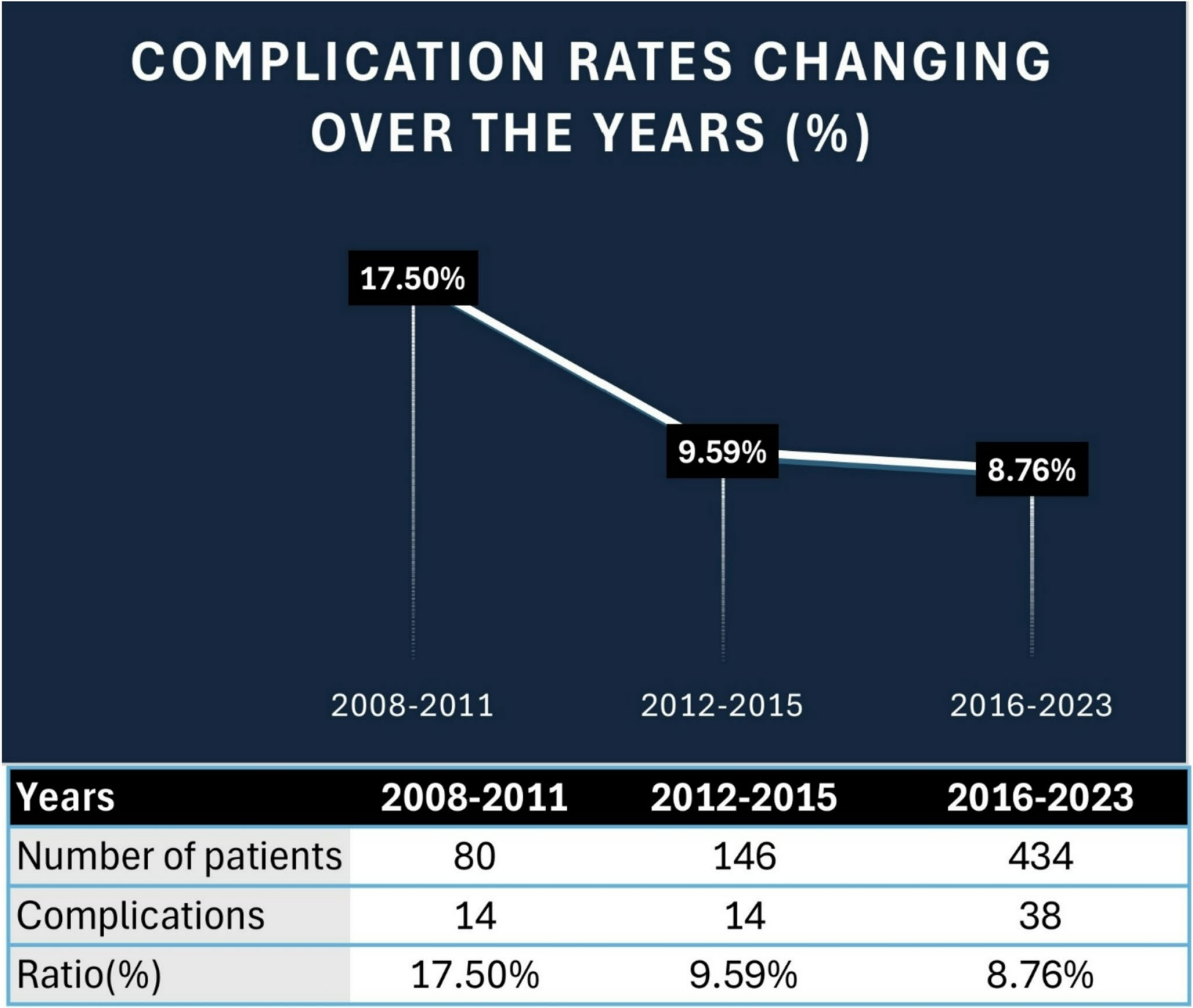
Changes in complication rates (%) over the years in treatments performed with the FDS technique

**Fig. 6:**
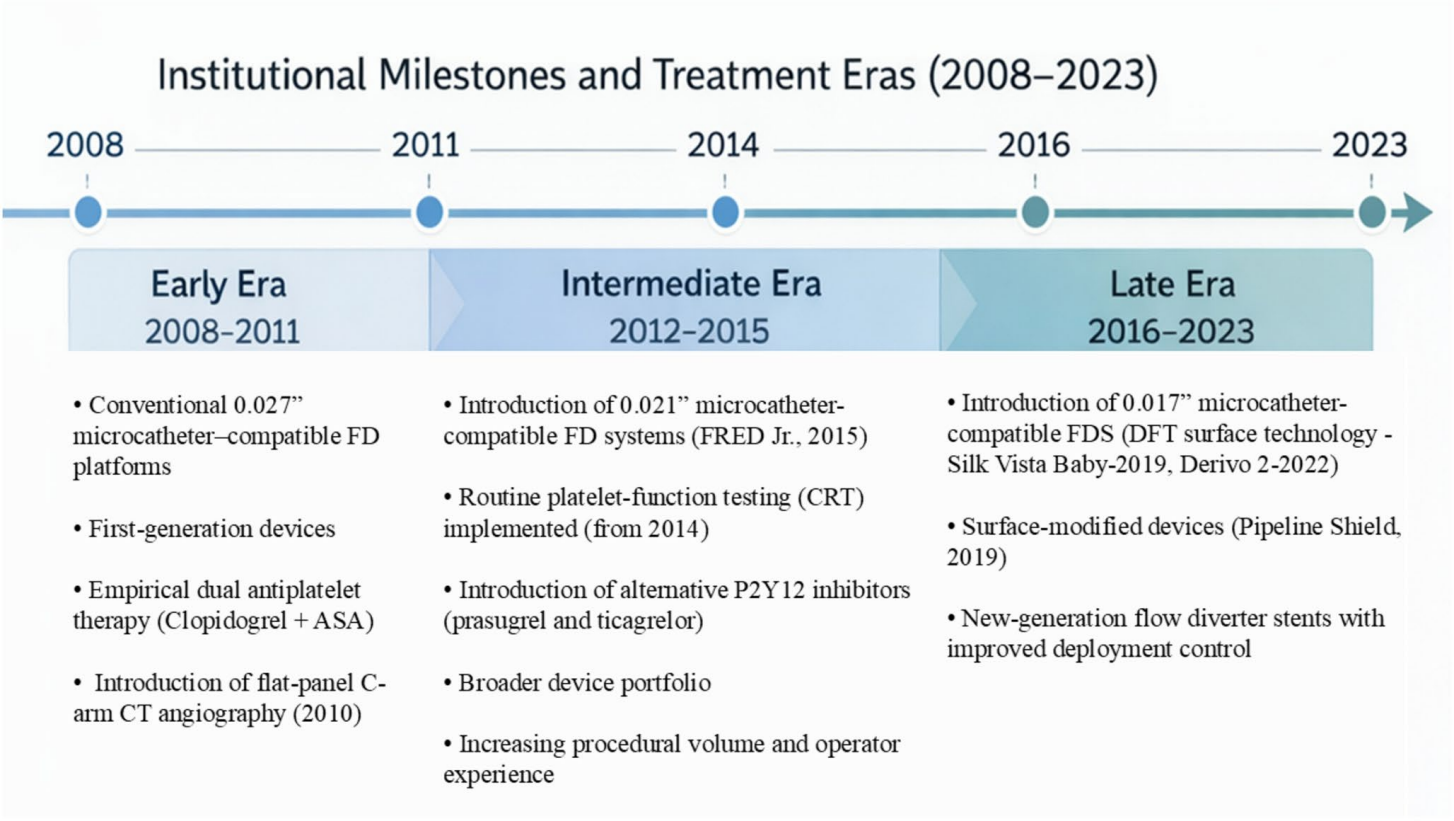
Institutional milestones and predefined treatment eras (2008–2023). The 15-year study period was stratified into three predefined eras (Early: 2008–2011; Intermediate: 2012–2015; Late: 2016–2023) reflecting progressive changes in device technology, delivery platform miniaturization (0.027” → 0.021” → 0.017” microcatheter compatibility), imaging protocols, and antiplatelet management strategies. Key milestones included the introduction of flat-panel C-arm CT (2010), routine platelet-function testing (2014), miniaturized flow diverter systems (FRED Jr., 2015; Silk Vista Baby, 2019), and surface-modified devices (Pipeline Shield, 2019; Derivo 2 with DFT technology, 2022). These eras were defined a priori for descriptive stratification of outcomes and do not imply causal relationships between technological milestones and complication rates

In our study, the overall patient-based complication rate was 9.70%, aligning with the ATENA trial, where stent- and balloon-assisted treatments had an 11.7% complication rate, compared to 10.8% for primary coiling [57]. This suggests similar complication rates between FDSs and remodeling techniques, though coiling alone shows slightly lower risks. A numerical decrease in complication rates was observed across treatment eras, from 17.50% in the early period to 9.59% in the intermediate phase and 8.76% in the later years (Fig. 5). These temporal trends paralleled progressive refinements in device technology, delivery platform miniaturization (0.027” → 0.021” → 0.017”), integration of flat-panel C-arm CT imaging (since 2010), routine platelet-function testing (from 2014), and the introduction of surface-modified flow diverters, as summarized in Fig. 6. The era-based stratification reflects this cumulative institutional evolution over time. Future multicenter studies will refine complication management and optimize treatment protocols.

**Fig. 7:**
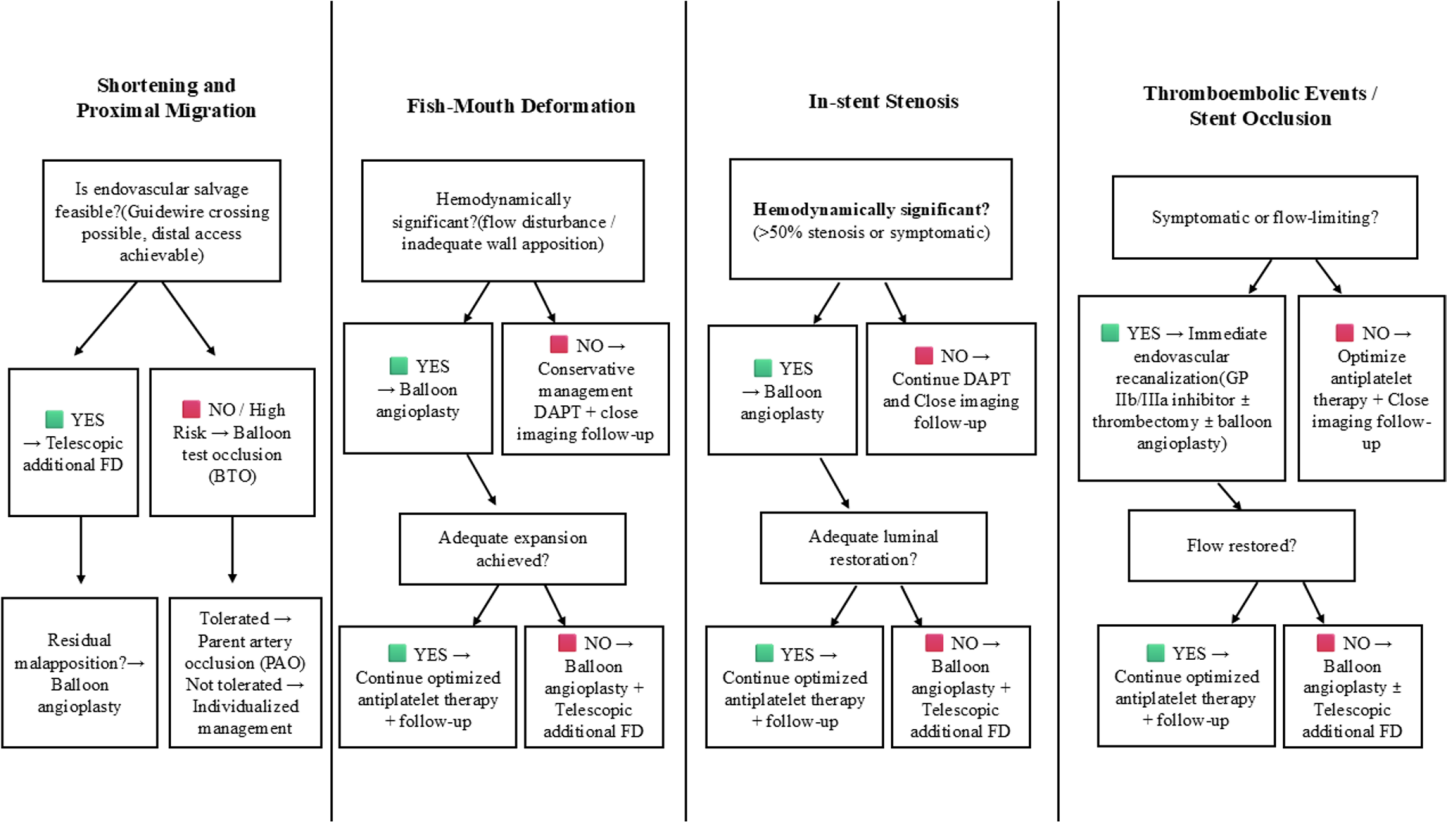
Practical management and bailout algorithms for flow-diverter–related complications. Schematic overview of complication-specific diagnostic evaluation and secondary (bailout) treatment pathways derived from our 15-year single-center experience. These algorithms reflect institutional management strategies rather than formal guideline recommendations

**Fig. 8:**
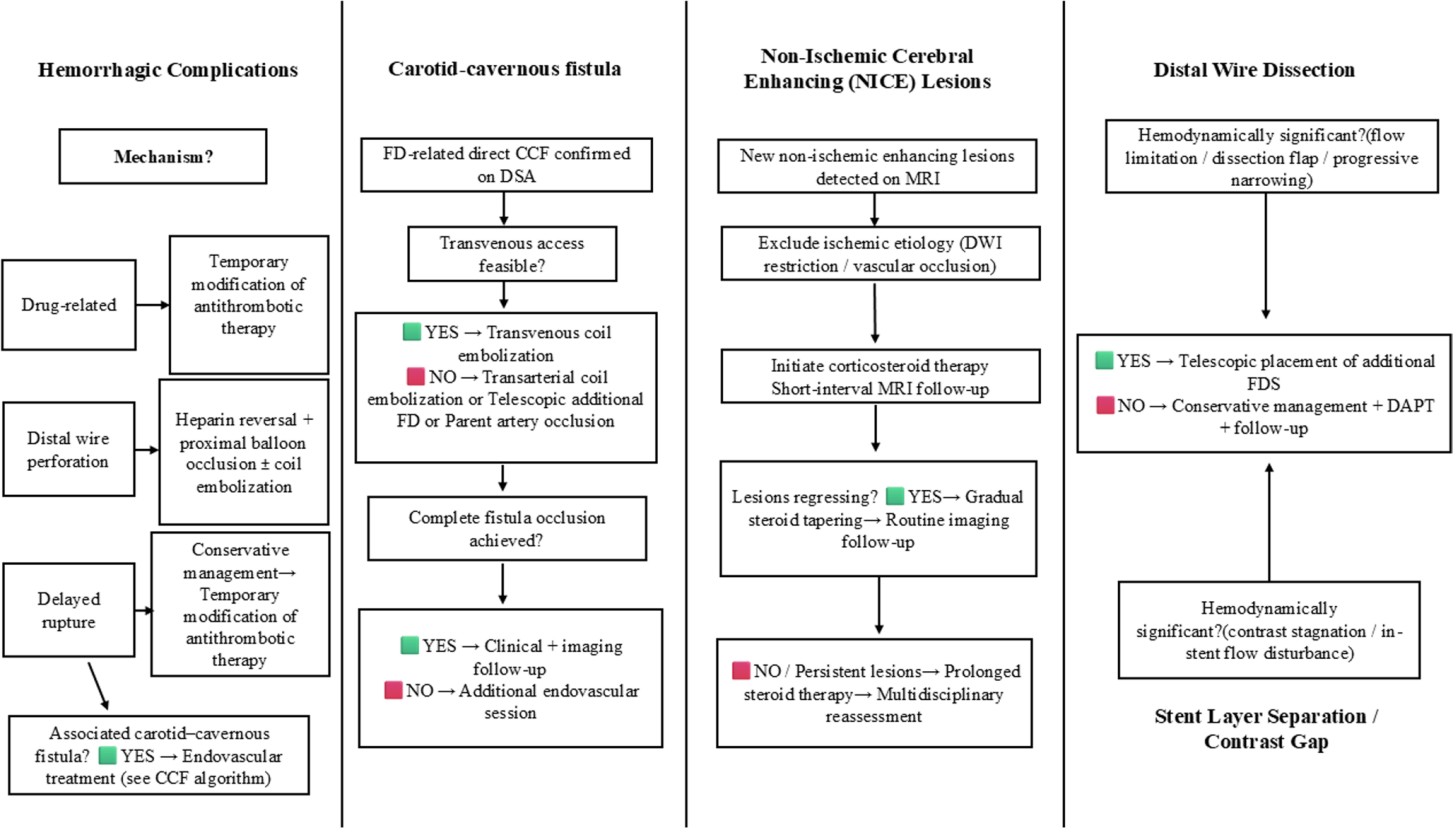
Practical management and bailout algorithms for flow-diverter–related complications. Schematic overview of complication-specific diagnostic evaluation and secondary (bailout) treatment pathways derived from our 15-year single-center experience. These algorithms reflect institutional management strategies rather than formal guideline recommendations

Based on our cumulative 15-year institutional experience, we developed structured, complication-specific management algorithms to summarize commonly used bailout pathways in device-related adverse events. These pragmatic frameworks are intended to enhance transparency and reproducibility of our institutional practice and are presented for educational purposes rather than as guideline-level recommendations (Figs. 7 and 8).

### Limitations

The major limitation of the study is the single-center retrospective design. Furthermore, while flow-diverter stents function with the same concept, they show considerable technical differences in terms of metal content, design, and deployment, which may influence occlusion and complication rates. Since various FDS devices and several different generations of these devices have been used in our clinic over the last 15 years, we believe that making a statistical comparison between these stents is not appropriate due to data heterogeneity. In addition, braid deformation and in-stent stenosis were assessed retrospectively without standardized grading, and subtle or asymptomatic changes were likely under-recognized; therefore, we refrained from reporting overall incidence rates for these findings and focused on the subgroup of patients who required secondary endovascular treatment.

## Conclusion

Flow diversion represents an effective reconstructive strategy for intracranial aneurysms, demonstrating durable long-term occlusion in a large single-center cohort. Complication patterns and management strategies evolved over time; however, the retrospective design and concurrent changes in devices and institutional protocols preclude definitive causal inferences. Our experience may serve as a reference framework for complication recognition and bailout management in contemporary practice.

## Data Availability

No datasets were generated or analysed during the current study.
